# Preparation and Culture of Myogenic Precursor Cells/Primary Myoblasts from Skeletal Muscle of Adult and Aged Humans

**DOI:** 10.3791/55047

**Published:** 2017-02-16

**Authors:** Ana Soriano-Arroquia, Peter D. Clegg, Andrew P. Molloy, Katarzyna Goljanek-Whysall

**Affiliations:** ^1^Institute of Ageing and Chronic Disease, University of Liverpool; ^2^Aintree University Hospital

**Keywords:** Developmental Biology, Issue 120, primary myoblasts, muscle, ageing, regeneration, microRNA, human

## Abstract

Skeletal muscle homeostasis depends on muscle growth (hypertrophy), atrophy and regeneration. During ageing and in several diseases, muscle wasting occurs. Loss of muscle mass and function is associated with muscle fiber type atrophy, fiber type switching, defective muscle regeneration associated with dysfunction of satellite cells, muscle stem cells, and other pathophysiological processes. These changes are associated with changes in intracellular as well as local and systemic niches. In addition to most commonly used rodent models of muscle ageing, there is a need to study muscle homeostasis and wasting using human models, which due to ethical implications, consist predominantly of *in vitro* cultures. Despite the wide use of human Myogenic Progenitor Cells (MPCs) and primary myoblasts in myogenesis, there is limited data on using human primary myoblast and myotube cultures to study molecular mechanisms regulating different aspects of age-associated muscle wasting, aiding in the validation of mechanisms of ageing proposed in rodent muscle. The use of human MPCs, primary myoblasts and myotubes isolated from adult and aged people, provides a physiologically relevant model of molecular mechanisms of processes associated with muscle growth, atrophy and regeneration. Here we describe in detail a robust, inexpensive, reproducible and efficient protocol for the isolation and maintenance of human MPCs and their progeny — myoblasts and myotubes from human muscle samples using enzymatic digestion. Furthermore, we have determined the passage number at which primary myoblasts from adult and aged people undergo senescence in an *in vitro* culture. Finally, we show the ability to transfect these myoblasts and the ability to characterize their proliferative and differentiation capacity and propose their suitability for performing functional studies of molecular mechanisms of myogenesis and muscle wasting *in vitro*.

**Figure:**
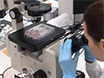


## Introduction

Disease- and age-related progressive loss of skeletal muscle mass and function results in frailty, decline in strength and decrease in quality of life of aged people. Skeletal muscle accounts for approximately 40% body mass^1^. During ageing and disease, progressive atrophy of individual myofibers and reduction of muscle quality due to the infiltration of fat and fibrosis occurs^1^,^2^,^3^,^4^,^5^,^6^.**Recently, it has been proposed that species-specific differences in ageing of skeletal muscle occur, specifically that muscle fiber loss occurring in rodents, may not occur in humans^7^. Nevertheless, the remaining muscle fibers of aged mammals are characterized by increased susceptibility to damage and impaired regeneration^8^.**Adult muscle repair and maintenance is mediated by satellite cells^9^,^10^. Upon muscle injury and other relevant cues, satellite cells become activated and proliferate. A subset of the cells returns to the quiescent state and the remainder progresses into myoblasts (Myogenic Progenitor Cells - MPCs). These contribute to repair of the existing myofiber^11^. The functionality of satellite cells determines the success of muscle regeneration and the changes in satellite cell availability with ageing have been demonstrated^12^,^13^,^14^,^15^. Moreover, satellite cells from the muscle of old humans and rodents show a transcriptional profile switch and reduced regenerative potential^16^,^17^,^18^,^19^. Satellite cells of muscle from old mice and humans have also been shown to undergo senescence resulting in their reduced functionality^20^.

The most established cell line enabling the study of muscle homeostasis is murine C2C12 cell line^21^. A significant amount of studies have also used murine primary myoblasts^22^. These cultures have led to a significant understanding of murine and vertebrate myogenesis as well as muscle regeneration, myotube/myofiber atrophy, and hypertrophy processes occurring during muscle disease and ageing^23^,^24^,^25^,^26^. More recently, several groups have described using human primary myoblasts to study myogenesis and muscle ageing. However, there is lack of consensus with regards to differences between primary myoblasts isolated from the muscle of adult and aged humans^27^,^28^,^29^,^30^,^31^. Despite differences characterized in the systemic and local environment occurring during development, ageing and disease 6,32,33,34, *in vitro* myoblast and myotube cultures remain the most accessible tools for studying molecular mechanisms associated with muscle development, growth and atrophy. Additionally, these studies provide not only a robust, but also a relatively quick, inexpensive and high-throughput *in vitro* tool. Moreover, ethical implications associated with studies of human muscles mean that for functional experiments involving manipulations of gene expression*, in vitro* human myoblast and myotube cultures remain the only alternative available to vertebrate model organisms.

Here, we show a simple experimental protocol for robust, inexpensive, and reproducible isolation of primary myoblasts, or MPCs, from the muscle of adult and aged people and describe standardized conditions of *in vitro* culture (**Figure 1**). As primary cultures from muscle usually contain fibroblasts in addition to myoblasts, we recommend a preplating step aiming at improved purity and quality of primary myoblasts. To summarize, we have established a protocol allowing for efficient and reproducible isolation, culture and functional studies of enriched and functional MPCs/primary myoblasts from skeletal muscle of adult and aged people.

## Protocol

All experimentation involving human tissue described herein was approved in advance by University of Liverpool, University Hospital Aintree Hospital and South West Wales Research Ethics Committee (Approval No: 13/WA/0374) and experiments were performed according to good practice guidance. The University of Liverpool acted as the ethics sponsor for this study. All the donors have given informed consent for the enrolment of this study. The muscles were isolated from people (BMI <25): adult: 30 ±2.8 years old and aged: 69 ±5 years old.

### 1. Preparation for Culture

1. **Coating of culture surfaces with laminin**
  1. Prepare a working solution of 10 µg/mL of laminin in 1x DPBS (Dulbecco's Phosphate Buffered Saline).
  2. Pipette a minimum amount of laminin solution to completely cover the surface onto which cells are going to be plated (**Table 1**). Incubate the culture dish at least 30 min in a humidified 37 °C, 5% CO_2_ incubator before plating the cells. Handle laminin carefully, avoiding the use of vortex. The working solution of 10 µg/mL laminin diluted in DPBS can be stored at 4 °C and re-used several times.
  3. Use a 60 mm (20 cm^2^) Petri dish or 2 wells in a 6-well plate (2 x 10 cm^2^) per ~18 - 19 mg of skeletal muscle to plate the cells (5.50 x 10^4^ cells in total). Perform cell counting at all times when plating cells for functional studies. NOTE: Samples were originally obtained from foot surgeries (extensor digitorum brevis, tibialis anterior or abductor halluces muscles) of female patients (adult: 30 ±2.8 years old, aged: 69 ±5 years old, BMI <25).
2. **Preparation of enzymatic solution**
  1. Prepare 250 mM CaCl_2_ working solution: 277 mg of stock CaCl_2_ in 10 mL 1x DPBS. Filter the solution with a 0.2 µm filter membrane and store at 4 °C.
  2. Prepare a working solution of 1.5 U/mL of collagenase D, 2.4 U/mL of Dispase II and 2.5 mM CaCl_2_ in serum-free DMEM (Dulbecco's Modified Eagle's Medium) (**Table 2**).
  3. Mix well and filter the enzymatic solution through a 0.2 µm filter membrane for sterilization. Prepare the enzymatic solution in advance and freeze down (-20 °C) in aliquots for future use.

### 2. Tissue Digestion: Mechanical and Enzymatic Dissociation

1. Following sample collection, keep muscle at 4 °C in DPBS until digestion. Prepare a minimum volume of 2 mL of collagenase-dispase-CaCl_2_ solution per 18 - 19 mg of muscle tissue and warm it to 37 °C before tissue dissociation.
2. Immerse the muscle biopsy briefly in 70% ethanol, wash with fresh DPBS and place the tissue on a new Petri dish with 1 mL of enzymatic solution.
3. Discard as much fibrotic and fat tissue as possible and tear the muscle quickly but gently into small but distinguishable pieces (approximately >0.5 mm^2^) with sterile scissors or a surgical scalpel (blade No. 10).
4. Transfer the sample into a 50 mL tube with the remaining 1 mL of the collagenase-dispase-CaCl_2_.
5. Incubate the tissue at 37 °C up to 30 - 40 min. Move the muscle tissue by gently agitating the tube every 5 - 10 min.
6. Coat the pipettes with media to avoid the adhesion of the released cells to the plastic walls of the pipettes. Add 2 volumes of sterile Growth Medium,* e.g.,* 4 mL of DMEM 20% FBS (Fetal Bovine Serum), 1% L-glutamine and 1% P/S (Penicillin-Streptomycin), to stop the digestion.
7. Pipet up and down several times with a 5 mL pipette to help the release of the cells from the muscle fibers. NOTE: The sample is usually completely dissociated due to its small size. However, if fragments of muscle still remain, use a second incubation with the remaining pieces of muscle and with fresh enzymatic solution.
8. Filter the muscle solution through a 70 µm cell strainer over a 50 mL conical tube. Wash the remaining cells with more media and filter through the strainer.
9. Centrifuge at 443 x g for 5 min at RT to pellet the cells. Discard supernatant carefully.
10. Dissolve the pellet into F-12 media (Ham's F-12 Nutrient Mix), 20% FBS, 10% HS (Horse Serum), 1% P/S, 1% α-glutamine and 2.5 ng/mL of FGF-b (Recombinant human basic Fibroblast Growth Factor). Here, use 4 mL of media per 60 mm Petri dish.

### 3. Seeding of Cells

1. Collect the culture vessel previously coated with 10 µg/mL laminin from the incubator (section 1.1).
2. Carefully remove the excess of laminin from the culture dish, and avoid touching the surface (or it will disturb the protein structure). Wash the culture dish with DPBS (optional).
3. Plate the cells directly on the laminin coated vessel and incubate for 24 h in a humidified 37 °C, 5% CO_2_ incubator.
4. Visualize the cells under the bright-field microscope (100X total magnification) the following day. Round small cells attached to the surface and the remaining debris can be seen in the culture media.
5. Change the media to fresh F-12 media complemented with 20% FBS, 10% HS, 1% P/S, 1% α-glutamine and 2.5 ng/mL of FGF-b.

### 4. Culture and Passaging of Cells

1. Change the media every 2 - 3 d and split the cells as soon as groups of cells are visible under the microscope (100X total magnification, example in **Figure 2**, cells from aged people, passage 0, after 7 d) in order to avoid spontaneous differentiation.
2. At the first passage (P1), change the media to high-glucose DMEM complemented with 20% FBS, 10% HS, 1% P/S, 1% α-glutamine. Avoid using FGF-b from this point, as FGF is a potent mitogen and important factor at the beginning of the culture, but it can promote fibroblast overgrowth if used longer in culture.
3. For cell passaging:
  1. Remove the media and wash the cells twice with DPBS.
  2. Add a minimum volume of 0.25% EDTA-trypsin to cover the surface of the cells. Rock gently to ensure all the cells are covered by the detachment solution, incubate for 10 seconds at room temperature, and remove it.
  3. Incubate the cells in a humidified 37 °C, 5% CO_2_ incubator for 3 - 5 min. Tap gently and check under the bright light microscope (100X total magnification) that the cells are rounded but not completely detached from the surface. If no change is observed in the cells, incubate for 5 more min.
  4. Add 5 mL of Growth Medium (high-glucose DMEM complemented with 20% FBS, 10% HS, 1% P/S, 1% α-glutamine) to collect the cells, mix well and transfer the cells with the new media into a T75 flask. Wash the remaining cells repeating this step with another 5 mL of Growth Medium (10 mL in total for one T75 flask).
  5. To preplate (preferable at the first passage), incubate the cells in a humidified 37 °C, 5% CO_2_ incubator for 40 min. Collect the supernatant with the cells that did not attach and incubate them in a new T75 flask. This should enrich the culture in myoblasts, as most of the fibroblasts should have attached in the first flask.
4. Change the media every 2 - 3 d and split the cells 1 to 4 as soon as they reach 70% confluency for a maximum yield.
5. For differentiation, once the cells reached 70 - 80% confluency, change the culture medium to Differentiation Medium (DM): high-glucose DMEM complemented with 2% HS, 1% P/S, 1% α-glutamine. The cells should be differentiated within 5 - 7 d depending on the quality and purity of the myoblast culture.

### 5. Transfections Protocol

1. Seed 50,000 cells/well in a 12-well plate for MF 20, senescence and viability assays. Culture cells on cover slips or in a dish coated with laminin. For Ki67 staining, seed 25,000 cells/well in a 12-well plate.
2. For transfections, follow the manufacturer's procedure using 5 µL of transfection reagent, 100 nM of control microRNA mimic or inhibitor, 100 nM of microRNA mimic or 100 nM of microRNA inhibitor per well, with a total volume of 1 mL.
3. Change to Differentiation Medium 6 h after transfection (high-glucose DMEM complemented with 2% HS, 1% P/S, 1% α-glutamine). For proliferation experiments, stain the cells 2 d after transfection; for senescence, 7 d after transfection; and for MF20 staining, seven days after transfection.

### 6. Immunostaining of the Cells

1. Ki67, MyoD and MF 20 immunostaining
  1. Prepare block 1 (10% HS and 0.1% Triton-X in PBS) and block 2 (10% HS and 0.05% Triton-X in PBS) solutions. Use 500 µL of reagent per well for a 12-well plate. NOTE: Perform the following steps using a shaker for the incubation steps.
  2. Remove the media from the cells.
  3. Rinse the cells with PBS.
  4. Fix the cells with cold methanol for 10 min on a rocker.
  5. Remove the methanol from the cells and rinse 3x with PBS for 5 min each time.
  6. Add block 1 solution and incubate the cells on a rocker at RT for 1 h.
  7. Add primary antibody solution: for Ki67 staining, use Rabbit mAb to Ki67 (1:1,000 dilution in block 2); for MyoD, use MyoD1 (D8G3) XP Rabbit mAb (1:100 dilution in block 2); for MF 20 staining, use MYH1E (MF 20) primary antibody (DSHB, 1:1,000 dilution in block 2).
  8. Incubate the cells with the primary antibody for 1 h (RT) to O/N (4 °C) on a rocker.
  9. Collect the primary AB (it can be re-used several times if stored at 4 °C).
  10. Rinse the cells 3x with PBS, 5 min each time.
  11. Add the appropriate secondary antibody: for Ki67 or MyoD staining, goat anti-rabbit IgG (H+L) secondary antibody, Alexa Fluor 488 conjugate (1:1,000 dilution in PBS); for MF 20 staining, goat anti-mouse IgG (H+L) secondary antibody, Alexa Fluor 488 conjugate (1:1,000 dilution in PBS).
  12. Wrap the plate containing the cells and incubate secondary antibody for 2 h in the dark on a rocker at RT.
  13. Rinse the cells 3x with PBS for 5 min each time.
  14. Add DAPI solution (1:1,000 dilution in PBS) onto the cells and incubate on the rocker at RT for 5 - 10 min.
  15. Rinse the cells 3x with PBS for 5 min each time.
  16. Add 1 mL of fresh PBS.
  17. Mount the cells on the cover slips in mounting solution or seal the plate containing the cells in PBS with Parafilm to avoid evaporation.
  18. Store at 4 °C.
  19. Visualize the cells with the fluorescence microscope as soon as possible (no later than 2 weeks).
2. **Viability assay**
  1. Remove the media from the cells.
  2. Rinse the cells in PBS.
  3. Add 1:1,000 ethidium bromide and 1:1,000 acridine orange diluted in PBS. Caution: Take care and work within health and safety regulations — ethidium bromide is a carcinogen.
  4. Wrap the plate containing the cells in staining solution and incubate at RT on the rocker for 5 min.
  5. Take images of the cells with the fluorescence microscope: green channel for acridine orange; red channel for ethidium bromide.

## Representative Results

MPCs/primary myoblasts should be visible 24 h post seeding onto the laminin-coated surface (**Figure 2**). The cells should adopt a spindle-like shape and should express MyoD still in passage 4 (**Figure 1A, B**). Fibroblasts can be distinguished by their star-like morphology and lack of expression of MyoD (**Figure 1B, C**). Once the cells are attached on the following day, media should be replaced with fresh bFGF media. The culture media should be replaced every 48 h.

The representative results shown here and published data from our laboratory^22^ aim to support our isolation and culture protocol and demonstrate the different techniques that can be used for functional studies of human primary myoblasts. Myoblast proliferation can be studied using Ki67 immunostaining and viability using staining for cell viability assay (**Figure 3**). For differentiation, the culture media should be changed to differentiation media. Myotubes should form in 5 - 7 d and be myosin heavy chain positive (**Figure 3**). Note that the myotube formation may be less efficient when myoblasts are isolated from the muscle of aged people (**Figure 3**). Senescence (SA-β-galactosidase) staining can also be performed in order to establish the percentage of senescent cells in the culture (**Figure 3**). We observed that with longer cultures (**Figure 3**, passage 4), more myoblasts isolated from the muscle of aged people show senescence.

For functional studies, gene and microRNA expression can be manipulated using lipophilic transfection reagents-mediated delivery of expression vectors, siRNAs, microRNA mimics and antimiRs (**Figure 4**). This allows for 40 - 70% transfection efficiency with gene/microRNA levels being up- or down-regulated within a physiological range (**Figure 4**;^22^).

**Table:** 

**Culture vessel**	**Approx. Area (per well)**	**Volume of 10 µg/mL laminin**
35 mm dish	10 cm^2^	1 mL
60 mm dish	20 cm^2^	2 mL
100 mm dish	60 cm^2^	4 mL
24-well plate	2 cm^2^	200 µL/well
12-well plate	4 cm^2^	500 µL/well
6-well plate	10 cm^2^	1 mL/well
T25	25 cm^2^	3 mL
T75	75 cm^2^	5 mL

**Table 1**. **Recommended Minimum Volumes of Laminin-DPBS Solution (10 µg/mL) for Coating Culture Surface.**

**Table:** 

	**Specific activity/Molar mass**	**Final Concentration**	**Mass or volume needed for 10 mL solution**
**Collagenase D**	0.15 U/mg	10 mg/mL (1.5 U/mL)	100 mg
**Dispase II**	0.5 U/mg	4.8 mg/mL (2.4 U/mL)	48 mg
**250 mM CaCl_2_**	110.98 g/mol	2.5 mM	100 µL

**Table 2. Enzyme Preparation for Muscle Digestion.**


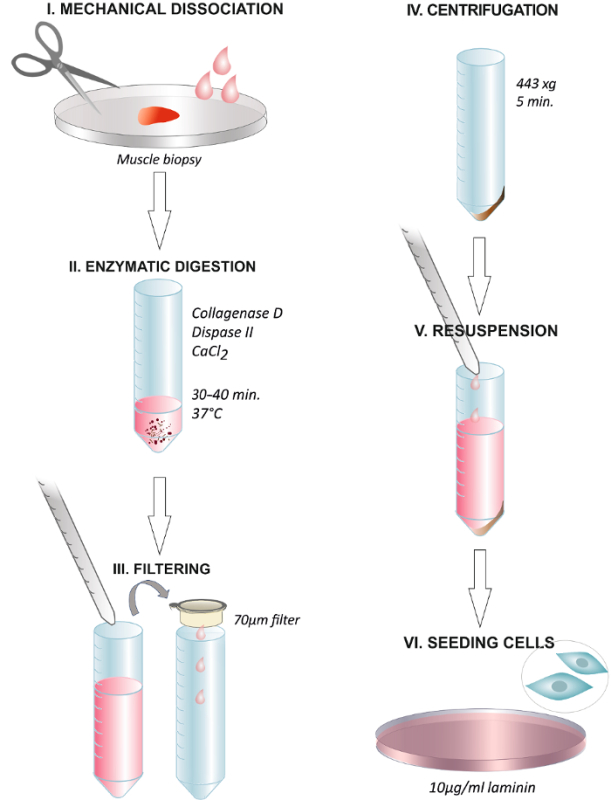
**Figure 1. Graphical Abstract Summarizing the Steps of the Protocol. **Dissociation of the human muscle biopsy with scissors or with a surgical scalpel (**I**). Incubation with the enzymatic solution at 37 °C for 30 - 40 min (**II**). End of the digestion through adding growth media and filtering the solution through a 70 µm membrane filter into a centrifuge tube (**III**). Centrifugation at 443 x g for 5 min (**IV**). Discarding the supernatant and resuspending in growth media containing 2.5 ng/mL FGF (**V**). Plating the cells on a dish coated with 10 µg/mL of laminin and changing the media after 24 h (**VI**). Please click here to view a larger version of this figure.


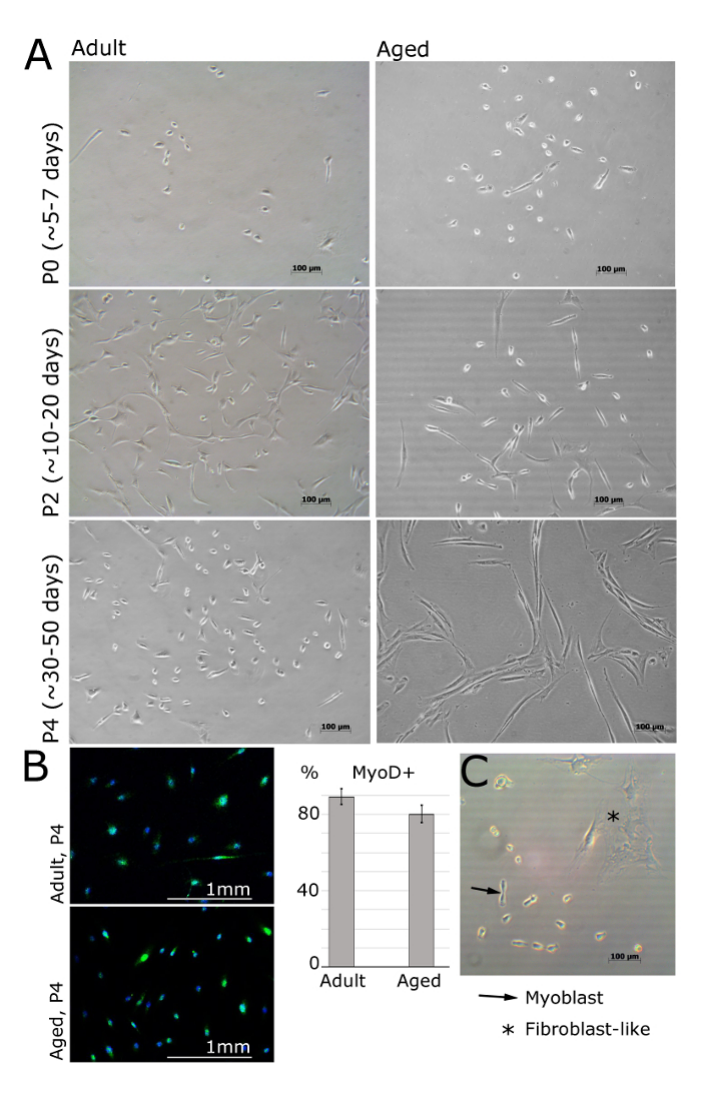
**Figure 2. Myoblasts Isolated from Muscle of Adult and Aged Humans at Different Passages. A**. Images represent myoblasts isolated from extensor digitorum brevis, tibialis anterior or abductor halluces muscles of female patients (adult: 30 ± 2.8 years old, aged: 69 ± 5 years old, BMI<25). At passage 0 and after 5 days of being plated, cells are still round and small, but visible under the bright light microscope (**A**). Myoblasts will then adopt an elongated shape, like shown at passage 2 (**A**). MyoD is expressed in myoblasts but not in fibroblasts (**B**). Quantification of MyoD-positive cells is shown; error bars show standard deviation; n = 3 (**B**). Representative image demonstrating the differences between myoblast and fibroblast morphology (**C**). Please click here to view a larger version of this figure.


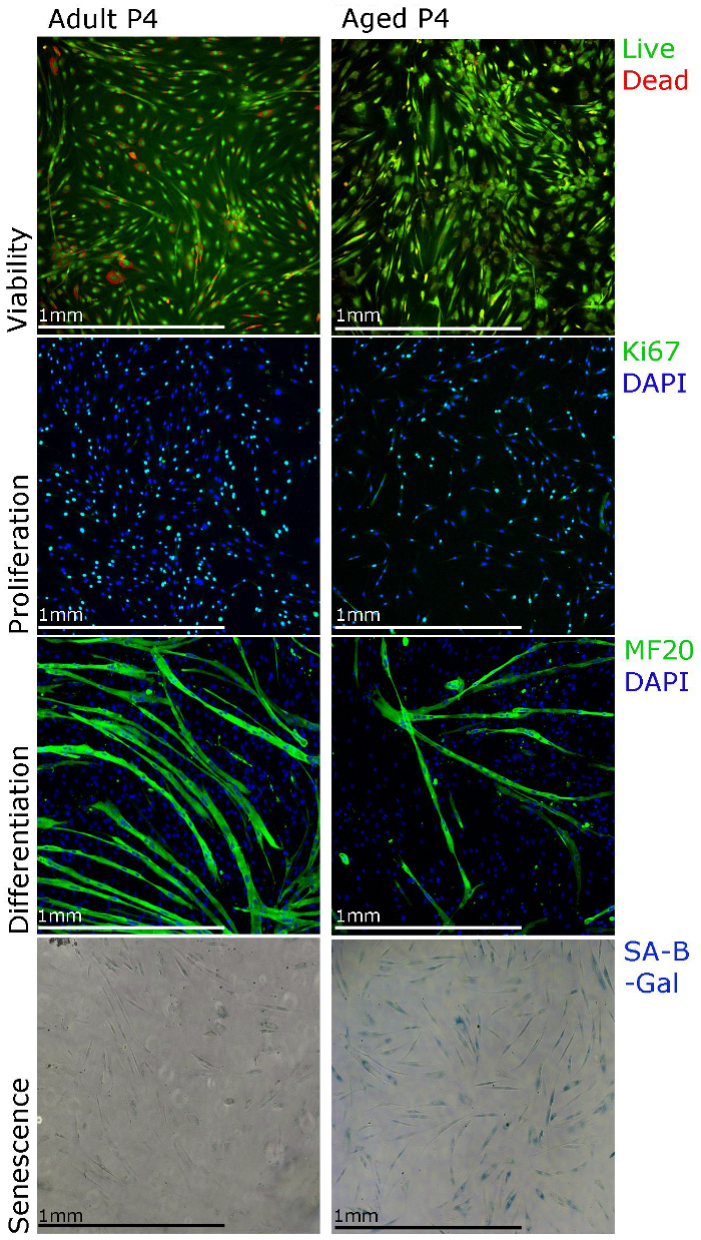
**Figure 3. Human Primary Myoblasts Can Be Characterized Using Different Staining Techniques. **Viability of the cells can be visualized using staining for cell viability assays, proliferation can be assessed using Ki67 immunostaining, differentiation can be assessed using MF20 (myosin heavy chain) immunostaining and senescence can be visualized using senescence associated beta galactosidase (SA-β-galactosidase) staining. Please click here to view a larger version of this figure.


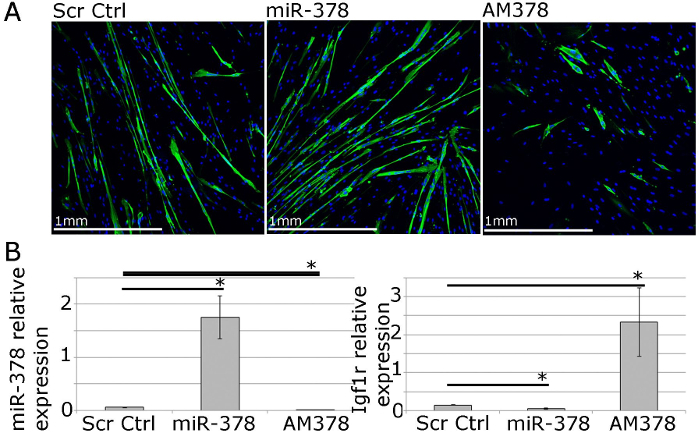
**Figure 4. Human Primary Myoblasts Can Be Used for Functional Studies of Muscle Homeostasis *In Vitro*. A.** MF20 (myosin heavy chain) immunostaining of differentiated primary myotubes from adult humans showing the effects of overexpression or inhibition of miR-378 on myotube size and number. **B.** qPCR showing relative expression of miR-378 and Igf1r, validated miR-378 target gene, following miR-378 overexpression or inhibition in human primary myoblasts. Expression relative to Rnu-6 and β-2-microglobulin, respectively, is shown. Error bars show SEM; n = 3, * - p<0.05, Student T test. Please click here to view a larger version of this figure.

## Discussion

Here, we present a simple, robust, inexpensive, reproducible and efficient method of isolating muscle progenitor cells/primary myoblasts from adult and aged humans from extensor digitorium brevis, tibialis anterior or abductor halluces muscles. This protocol aims to allow studies using human primary myoblasts from adult and aged humans, especially when more sophisticated methods, such as FACS- or MACS-sorting, are not possible or not practical.

The isolation method presented in this manuscript takes approximately 2 hours. During muscle isolation, muscle was washed in 70% ethanol in order to avoid contamination. Prior to enzymatic dissociation of the muscle, it is important to cut the muscle into small but visible pieces, and avoid cell damage from too much mincing. The digestion results in the dissociation of myofibers and the release of satellite cells and myogenic precursor cells. In our case for ~20 mg of skeletal muscle, one 60 mm (20 cm^2^) Petri dish was the most appropriate surface area for harvesting the cells. Cells plated onto a larger surface showed reduced proliferation, whereas cells plated onto a smaller surface showed increased cell death and agglutination.

Upon isolation, the cells were cultured and expanded on laminin-covered plates. The use of non-coated surfaces tended to decrease the success of the isolation. For this reason, cells can be preferably harvested on a pre-coated surface directly after isolation. Fibroblasts-enriched cultures will predominate rather than myoblasts-derived cells if the cells are harvested on a non-coated surface directly after isolation. Apart from laminin, the use of other cell attachment solutions such as Matrigel and collagen-based reagents can be used. Coating solutions may include growth factors and other compounds that will promote cell growth, but these could alter the cell behavior and therefore the experimental results. In our experience, 10 µg/mL laminin is the optimum concentration and appropriate coating reagent for satellite cells and myoblast attachment and proliferation as it lacks any growth factor or other complements. Moreover, laminin is naturally present in the basal lamina, directly linked to the sarcolemma, which plays a key function in satellite cell attachment and migration through the skeletal muscle fiber.

The supplements of the culture media may also have a detrimental influence on the behavior of the primary myoblast. For example growth factor groups, such as FGFs or IGFs, have pleiotropic effects on primary myoblast cultures, with FGF-2 controlling both mitogenic and programmed cell death response^31^. It is therefore necessary to rigorously control the culture conditions, especially because the differences in the behavior of primary myoblasts isolated from muscle of adult and aged people are very likely to be due to the purity of the cultures and the likelihood of fibroblasts overrunning the myoblasts in culture during long-term cultures^35^. We have used 1-hour pre-plating of the cells during the first splitting onto a non-coated surface in order to decrease the contamination of the cultures with fibroblasts.

The method we describe is appropriate for isolating myogenic progenitor cells from the muscles of both adult and aged humans. The isolated cell consists of a representative myogenic population of cells as indicated by a high percentage of myogenic cells (MyoD expression and myogenic properties visualized by MF20 immunostaining in **Figures 1 **and** 2**) and can be used as an *in vitro* model for functional studies of processes associated with muscle homeostasis.

Previous studies have characterized the isolation and differences in properties, or the lack thereof, of human primary myoblasts from adult and aged people^6^,^20^,^27^,^28^,^29^,^30^,^31^,^35^,^3^,^6^,^37^,^38^. The existence of geriatric and/or non-functional human MPCs has been demonstrated^6^,^20^,^22^. However, no difference in the behavior of freshly isolated human MPCs has also been shown^27^. Our protocol allows for the isolation of primary myoblasts that at least partially retain their phenotype, such as reduced proliferative potential or senescence of primary myoblasts isolated from muscle of aged people and permits the use of these cells for functional studies of molecular mechanisms of muscle homeostasis during ageing^22^.

The primary myoblasts isolated using the method described here can be used not only for myogenic differentiation studies but also to investigate intracellular changes, such as changes in gene expression occurring in human myogenic precursor cells during ageing. However, changes that occur in cells during prolonged *ex vivo* culture need to be considered when analyzing phenotypic and genotypic changes occurring during ageing. We recommend using freshly isolated cells for this purpose.

Moreover, the primary myoblast culture method described here allows for expansion and relatively long-term culture of human primary myoblasts, allowing for robust *in vitro* functional studies. We have previously shown that myogenic progenitor cells isolated using our method can be used for both expression profiling and functional studies of processes associated with muscle ageing^22^. This method is also applicable to the muscles of adult and old rodents and allows for isolation of an enriched culture of myoblasts that can be used for profiling genetic and epigenetic changes during ageing and functional studies^22^. The limitations of this method include the use of, to some degree, mixed population of cells rather than a pure population of satellite cells, which can be obtained using more sophisticated published methods^6^,^28^,^29^,^39^,^40^,^41^,^42^,^43^.

We present a simplified, affordable, and reproducible protocol for the isolation of primary myoblasts cells from adult and aged humans. In our experience, the available, more sophisticated methods of isolation and culture of human primary myoblasts (such as MACS- or FACS-sorted satellite cells) are ideal for some types of studies, such as profiling transcriptomic or proteomic changes in the cells. However, these methods are expensive, require at least some level of expertise, and may prove difficult due to the low proliferative rate of pure primary myoblast cultures and fibroblasts overgrowing myoblasts.

We present a reproducible protocol that permits the simple isolation and culture of human primary myoblasts for use in functional studies. Additionally, we propose the use of laminin^42^ and the limited use of bFGF as key factors for a successful culture^44^. We also propose avoiding the stress generated by centrifugation when splitting the cells and one pre-plating step at the first passage^45^. To summarize, we have optimized an efficient protocol for isolation and culture of primary myoblasts/MPCs from the muscles of adult and aged humans that is also applicable to the muscles of rodents and enables expression and functional studies of muscle homeostasis.

## Disclosures

There is nothing to disclose.
